# Electrochemical tuning of the activity and structure of a copper-cobalt micro-nano film on a gold electrode, and its application to the determination of glucose and of Chemical Oxygen Demand

**DOI:** 10.1007/s00604-014-1353-z

**Published:** 2014-09-17

**Authors:** Jinqi Wang, Na Yao, Mei Li, Jia Hu, Jianwei Chen, Qiaoling Hao, Kangbing Wu, Yikai Zhou

**Affiliations:** 1 MOE Key Lab of Environment and Health, School of Public Health, Tongji Medical College, Huazhong University of Science and Technology, Wuhan, 430030 China; 2 School of Chemistry and Chemical Engineering, Huazhong University of Science and Technology, Wuhan, 430074 China

**Keywords:** Chemical oxygen demand (COD), Electrochemical sensor, Micro-nano Cu-Co, Gold electrode, Electro deposition

## Abstract

Micro-nano structured Cu-Co was in situ fabricated on the surface of a gold electrode via electrochemical reduction of CuCl_2_ and Co(NO_3_)_2_. It is shown that the shape of the particles can be controlled by variation of deposition current, deposition time, pH value and the ratio of Cu(II) and Co(II) ions. If prepared under current of −200 μA in 0.1 M, pH 4.0 acetate buffer solution, the film possesses high catalytic activity towards the electrochemical oxidation of glucose at a largely increased oxidation current compared to a non-modified surface. The electrochemical activity of this sensor can be easily tuned. Glucose is a standard compound for evaluating the chemical oxygen demand (COD), and we have therefore studied the application of the sensor to the determination of this parameter. Under optimized conditions, the sensor has linear response to glucose in the 1.92-768 mg L^−1^ concentration range, and the detection limit is 0.609 mg L^−1^ (at an S/N ratio of 3). A large number of surface water samples was studied, and the results obtained by this method were found to be linearly correlated to those obtained by the dichromate method (r = 0.995; *n* = 33).

**Electronic supplementary material:**

The online version of this article (doi:10.1007/s00604-014-1353-z) contains supplementary material, which is available to authorized users.

**Graphical Abstract:**
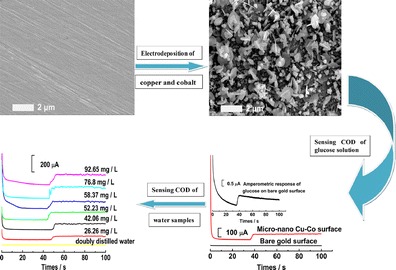
This study describes the facile synthesis of micro-nano Cu-Co by one-step electrodeposition of Cu(II) and Co(II) on gold electrode. The alloy composite exhibited excellent electrocatalytic activities, and was successfully applied on the COD determination of glucose and water samples.

## Introduction

Chemical oxygen demand (COD) is defined as the number of oxygen equivalents consumed in the oxidation of organic compounds using strong oxidizing agents [1, 2]. However, the application of the standard determination method has often been limited by many intrinsic drawbacks, including time consuming process, complicated operation, consumption of expensive (Ag_2_SO_4_), highly corrosive (H_2_SO_4_) and toxic (Cr_2_O_7_^2−^ and HgSO_4_) reagents [3, 4]. Thus, numerous efforts have been made to develop simple and rapid analytical method for COD [5, 6]. Among them, the electrochemical method has considered as a promising method for COD detection due to its high sensitivity, short analysis time, low cost and handling convenience. In order to achieve high response signal, development of highly sensitive electrode material is crucial. So far, different materials were successfully used to construct sensing electrodes for COD. The electrocatalytic oxidation method utilized TiO_2_ nanorod [7],TiO_2_ film [8], Rh_2_O_3_/Ti [9], Ti/Sb-SnO_2_/PbO_2_ [10], AgO-CuO [11] and photocatalytic nano-ZnO/TiO_2_ [2] as sensors to oxidize organic compounds. However, the fabrication procedure was complicated and time-consuming compared with electrodeposition method. Boron-doped diamond (BDD) [12–14], nano-Pt [15] and Nano-PbO_2_ [16] have been reported with satisfied performance for COD determination. However, their application in practice was limited by the complicated fabrication process requiring special instrument leading to high cost of BDD and Pt. Nano-Cu [3, 17] and F-PbO_2_ [15] were also fabricated for COD determination. Nevertheless, the methods were incapable of widespread application due to their high detection limit.

Chemical oxygen demand (COD), a major indicator of organic pollution in waters, is widely recognized as one of the most important parameters for water quality [18–20]. Up to now, many countries, such as China and Japan, have accepted it as a national standard for organic pollution evaluation [21]. Although the overall reduction of COD is complicated, it was found that some simple reactivities are common and can be enhanced to improve the catalytic activity of sensing films. The typical reaction of COD reduction is presented as:

$$ {C}_y{H}_m{O}_j{N}_k+\left(y+\frac{m}{4}-\frac{j}{2}-\frac{3}{4}k\right){O}_2\to yC{O}_2+\left(\frac{m}{2}-\frac{3}{2}k\right){H}_2O+ kN{H}_3 $$

From this equation we could see that the reduction of COD includes one common progress: oxygen-oxygen band-breaking.

Mixing two or more metals to generate intermetallic compounds can result in a catalyst that has distinct properties from its monometallic components. The rich diversity of the compositions, structure and properties of intermetallic compounds has led to their widespread application in field of catalysis [22]. During the searching for abundant, inexpensive, and efficient electrocatalytic materials, researchers found that the addition of metals, which bind oxygen strongly, can lower oxygen binding to other metals and easily break the oxygen-oxygen bond and eventually improve their catalytic activity [23]. As known, Cu and Co are both metals that can bind oxygen strongly and tend to reduce the oxygen easily [24]. Then we could speculate that the alloy composition in Cu-Co sensing film may not only prevents Co from being corroded too rapidly [25], but also exhibits the synergistic effects on oxygen-oxygen band breaking, and ultimately enhance the response signal of COD detection.

In this work, micro-nano Cu-Co composite was in situ fabricated on the surface of gold electrode via electrochemical deposition using Co(NO_3_)_2_ and CuCl_2_ as the precursor. The Cu-Co composite was characterized by scanning electron microscopy (SEM), which revealed that this composite formed a film with a rough structure and a large surface area on gold electrode. Linear sweep voltammetry (LSV) was employed to illustrate the electrochemical oxidation of glucose in alkaline solution. The influence of electrodeposition parameters on glucose response signal was studied, and then a novel electrochemical sensor was developed. Compared with the reported sensors as shown in table 1, this new one exhibited higher sensitivity, higher Cl^−1^ tolerance and the detection limit was as low as 0.609 mg L^−1^ COD. Moreover, this new sensor was successfully applied to the determination of COD in surface water, which indicates that this micro-nano composite could be used as an electrocatalyst for the sensing and detection of COD in real water samples. The present work not only provided a sensitive electrochemical method for the determination of COD in surface water, but also demonstrated the promising applications of intermetallic micro-nano material composite as highly active sensing materials in high-performance electrochemical sensors.

**Table 1:** Comparison of electrochemical sensors for COD

Sensing material	Detection limit	Linearity range	Sensitivity	Tolerance of Cl^ − 1^	Detection Potential	Reference
	(mg L^-1^)	(mg L^ − 1^)	(μA/(mg L^ − 1^ COD))	(M)	(V)	
TiO2 nano-rod array	18.3	20–280	––	––	0.5	[7]
Cobalt oxide	1.1	1.7–170	1.00	0.02	0.8	[26]
F-PbO2	15	100–1200	0.000230	––	1.3	[15]
Cu/CuO	20.3	53–2801	0.472	––	––	[17]
Rh2O3/Ti	20.0	50–2000	0.0220	0.017	1.3	[9]
BDD^a^	1	2–175	0.0909	––	2.8	[27]
TiO2/Ti/TiO2-Pt	9.5	25–380	3.55	0.040	–	[28]
Cu	3.6	4.8–600	0.454	0.02	0.8	[3]
Pt	1.83	––	0.0260	––	0.45	[29]
Cu2O-TNTAs^b^	15	20–300	1.45	0.0056	0.3	[30]
Cu-Co	0.609	1.92–768	0.888	0.02	0.6	This work

^a^BDD: boron-doped diamond

^b^TNTAs: TiO2 nanotube arrays electrode

## Experimental

### Reagents

All chemicals were of analytical grade and used as received. Co(NO_3_)_2_, CuCl_2_, NaOH, K_2_Cr_2_O_7_, Ag_2_SO_4_, HgSO_4_, (NH_4_)_2_Fe(SO_4_)_2_, HCl, NaNO_3_, acetic acid and sodium acetate were purchased from the Sinopharm Group Chemical Reagent Co. Ltd. (China, http://en.reagent.com.cn/). Doubly distilled water was used throughout. Surface water samples were collected on-site from different lakes and rivers in Wuhan (China). Water samples were stored in dark at 4 °C and analyzed within 48 h.

### Instruments

Electrochemical measurements were performed on a CHI660D Electrochemical Workstation (Chenhua Instrument, Shanghai, China http://www.chinstr.com/) with a conventional three-electrode system. The working electrode is a micro-nano Cu-Co modified gold electrode, the reference electrode is a saturated calomel electrode (SCE), and the counter electrode is a platinum wire. Scanning electron microscopy (SEM) was performed with a Quanta 200 microscope (FEI Company, Netherlands, http://www.fei.com/).

### Preparation of micro-nano Cu-Co sensing film

Before electrodeposition, the gold electrode with diameter of 3 mm was polished with 0.05 μm alumina slurry, and then sonicated in doubly distilled water to give a clean surface. After that, micro-nano Cu-Co sensing film was formed on 3 mm gold electrode surface under −200 μA for 100 s in 0.1 M, in pH 4.0 acetate buffer solution containing 27 mM CuCl_2_, 3 mM Co(NO_3_)_2_ (vs. SCE). Finally, the resulting electrode was rinsed with re-distilled water to remove any adsorbed species. For comparison, gold electrode was also prepared following a similar procedure in the absence of Cu^2+^ and Co^2+^.

### Detection of COD using standard dichromate method

The dichromate method was used to measure COD value according to the National Standard of China (GB 11914–89). Water sample solution (10.0 mL) and 0.025 M K_2_Cr_2_O_7_ solution (5.0 mL) were added into a cuvette, and then refluxed for 2 h in a thermostat at 160 °C.

After that, the excess of dichromate was determined by titration using 0.005 M (NH_4_)_2_Fe(SO_4_)_2_ as the titrant. Finally, the value of COD was calculated after subtracting the blank value of doubly distilled water.

### Linear Sweep Voltammetry and amperometric detection of COD using micro-nano Cu-Co modified gold electrode

The linear sweep voltammetry (LSV) detection was employed to obtain the best reaction conditions. By detecting the oxidation current of glucose under various reaction conditions, the optimal operating conditions were determined.

The amperometric detection under well-stirred condition was used to measure the value of COD. The detected potential was at 0.6 V and the electrolyte was 0.1 M NaOH solution. The observed current on Cu-Co surface was allowed to reach a steady state, and then the standard sample or the real surface water sample was added. The net current increase was measured as the response current of COD. All experiments were carried out at ambient temperature.

## Results

### Current tuning morphology and electro catalytic activity of Cu-Co film

The influence of metal ion concentration, deposition medium and deposition time on the activity of Cu-Co was explored (see Supplementary Material). It’s found that Cu-Co sensing film prepared in 0.1 M, pH 4.0 acetate buffer with reduction time of 100 s using 27 mM Cu^2+^ and 3 mM Co^2+^ as precursor exhibits the highest electrochemical activity, and was employed throughout.

**Fig. 1:**
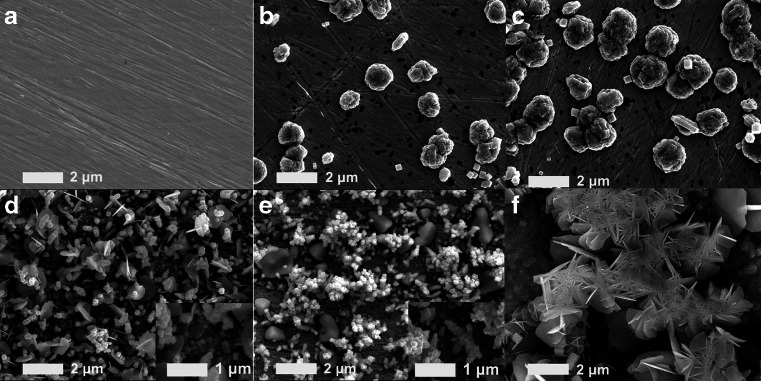
SEM images of bare gold surface (A) and the prepared Cu-Co at −100 μA (B), −150 μA (C), −200 μA (D), −250 μA (E), −300 μA (F) and micro-nano Cu at −200 μA (G)

**Fig. 2:**
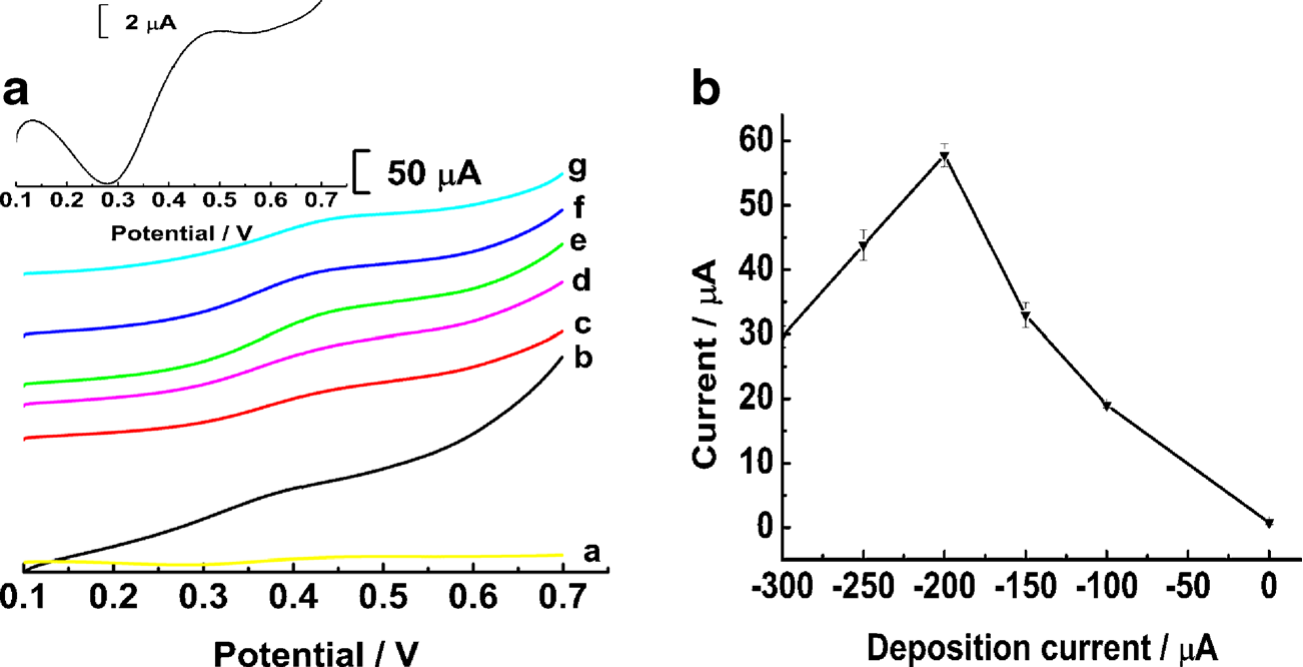
A: The LSV curves of 19.2 mg L^−1^ glucose on bare gold surface (a), micro-nano Cu at −200 μA (d) and the resulting micro-nano Cu-Co at −100 μA (b), −150 μA (c), −200 μA (e), −250 μA (f), and −300 μA (g). Insert plot: LSV curves of 19.2 mg L^−1^ glucose on bare gold surface. B: Variation of the oxidation peak current of 19.2 mg L^−1^ glucose on micro-nano Cu-Co as the reduction deposition current. The error bars represent the standard deviation of repetitive measurements (*n* = 3)

Electrochemical deposition has been widely used in preparing coatings with different functions because of its simple operation and low cost [31–33]. As the deposited current influences the morphology of prepared particles greatly, different deposited currents were applied and the morphology of prepared particles was characterized using 0.1 M, pH 4.0 acetate buffer with reduction time of 100 s. As shown in Fig. 1, when the deposited current was −100 and −150 μA (Fig. 1B, C), the electrodeposited Cu-Co films are consisting of few irregular flowerlike micro particles, besides, the size and density of them grows as time lasts. However, nothing appeared on the bare gold surface if reducing without Cu^2+^ and Co^2+^ (Fig. 1A). As the reduction current of −200 μA, micro and submicro needle-like, rod-like, flake-like and grain particles started to form on gold surface, among which numerous nanoparticles assembled on substrates with diameters ranging from 40 nm to 80 nm (Fig. 2D). This may be caused by the fact that deposition current of −200 μA enabled diversified types of crystal to nucleate and enabled crystal nucleation and growth to take place all over gold surface. For deposited current of −250 μA (Fig. 2E), the density of fully developed particles increases as well as their sizes (diameter and height) and nanoparticles start to conglomerate while few irregular particles, > 1 μm in diameter, distribute among them. The size and density of particles in the Cu-Co film increases even further when reduction current moved down to −300 μA (Fig. 2F), with giant flake-like particles appearing on the as-deposit film and nanoparticles hardly to find. Otherwise, sensing film deposited at −200 μA under the condition of 0.1 M, pH 4.0 acetate buffer, with 30 mM CuCl_2_ as the precursor, was also depicted. As shown in Fig. S5, a relatively thick coating consisted of numerous micro flake-like particles, accompanying with lots of submicro grain particles assembling on substrates. Apparently, the shape of Cu-Co film can be easily tuned by variation of the reduction current, at the current value of −200 μA, the gold surface was covered by sensing film that exhibiting high surface roughness and possessing numerous micro-nano particles, and therefore provided large response area and numerous active sites.

In order to elucidate the relationship between the electrochemical activity of micro-nano Cu-Co and its shape, the electrochemical oxidation of glucose taking place on the surface of micro-nano Cu-Co that prepared at different currents was studied using linear sweep voltammetry (LSV). Fig. 2A displays the LSV curves of 19.2 mg L^−1^ glucose in 0.1 M NaOH on the surface of different Cu-Co film, and Fig. 2B shows the variation of response signal with the deposition current. On the bare gold electrode, the oxidation peak current of glucose was hardly seen (curve a and the inset), suggesting that bare gold surface possessed much lower electrochemical activity to the oxidation of glucose. However, glucose was easily oxidized at the surface of micro-nano Cu-Co, and obvious oxidation peak appeared, indicating that micro-nano Cu-Co exhibits high activity to the oxidation of glucose. When the deposition current shifts from −100 to −200 μA (curve b-c, e), the response current of glucose increases remarkably. With further increasing the deposition current to −250 (curve f) and −300 μA (curve e), the response current of glucose decreases obviously. Clearly, the micro-nano Cu-Co that prepared at −200 μA exhibits highest response activity to the electrochemical oxidation of glucose. Besides, the LSV curves of glucose on the surface of electrodeposited copper film was also depicted (curve d) which is inferior than that on micro-nano Cu-Co film fabricated under the same condition (curve e). From the SEM images, it is observed that the particle size of micro-nano Cu-Co that prepared at −200 μA is smaller, and the arrangement was regular on the gold surface. There’s no doubt that the oxidation signal of glucose on the surface of micro-nano Cu-Co was much higher. This phenomenon reveals that micro-nano Cu-Co has unique properties which could enhance the oxidation signal of glucose largely.

### Detection of COD

It is very convenient to select some substances as the standard reagent for electrochemical detection of COD. Herein, glucose was used as the standard compound, and its oxidation current on micro-nano Cu-Co/gold surface was applied to evaluate the value of COD. The COD value of glucose standard solution was detected using conventional dichromate method. For glucose solution, the value of COD was measured to be 380.6 mg L^−1^ of O_2_ using the conventional dichromate method, which consists with the theoretic value of 384 mg L^−1^ of O_2_ [34].

In order to choose a suitable medium for the electrochemical detection of COD, the oxidation peak response of glucose in NaOH solution with different concentrations were studied using linear sweep voltammetry. Micro-nano Cu-Co was prepared at −200 μA for 100 s in 0.1 M, pH 4.0 acetate buffer containing 27 mM CuCl_2_ and 3 mM Co(NO_3_)_2_. Fig. S6 illustrates the effect of concentration of NaOH on the oxidation current of glucose solution with COD of 19.2 mg L^−1^. It is found that the oxidation current of glucose notably enhanced with NaOH concentration over the range from 0.05 to 0.1 M. When the concentration of NaOH is higher than 0.1 M, the oxidation signal of glucose begins to decrease. Thus 0.1 M NaOH was employed to obtain high electrochemical activity.

### Amperometric detection of COD

**Fig. 3:**
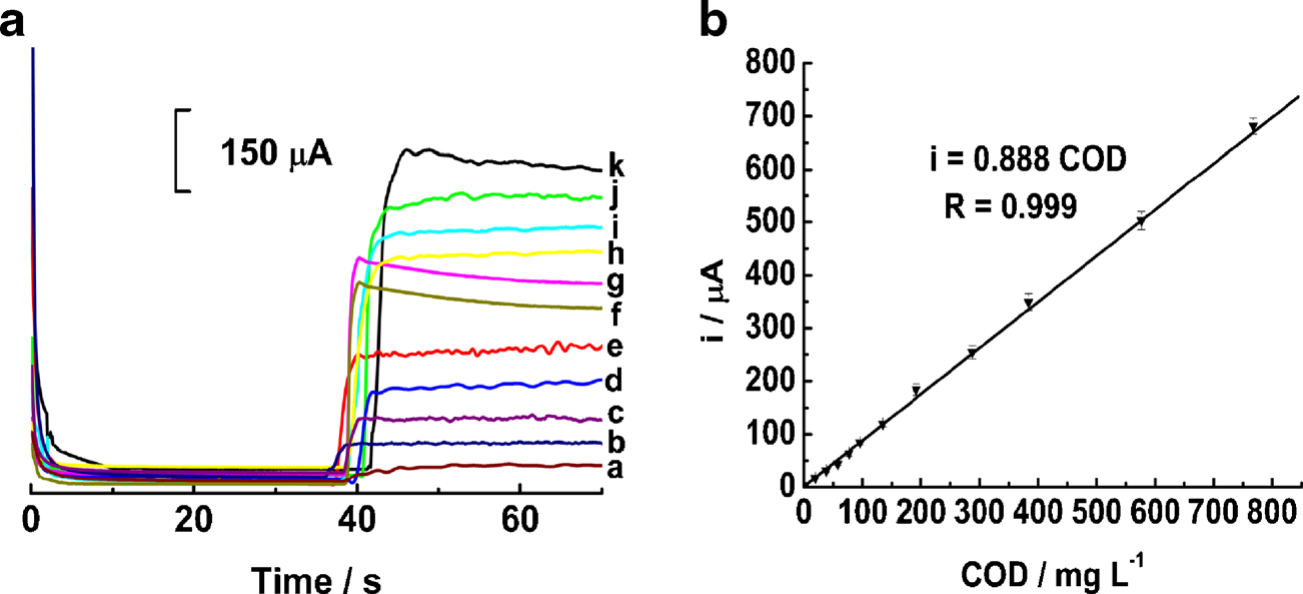
A: The amperometric response of micro-nano Cu-Co/gold electrode to glucose of different COD values in 0.1 M NaOH at 0.6 V. B: Calibration curve for COD values of glucose on micro-nano Cu-Co. Other conditions are as in Fig. 2

Amperometric detection, a simple and powerful analytical method, was used to detect the value of COD. Fig. 3A depicts the typical amperometric response of different-valued COD glucose solution on micro-nano Cu-Co surface in 0.1 M NaOH solution. When the oxidation potential is controlled at 0.6 V, the background current of micro-nano Cu-Co decreases dramatically during the first 5 s, and then attains a steady state at about 15 s. After addition of glucose solution with COD value of 19.2 mg L^−1^ at about 40 s, a slight response is observed (curve a). When adding glucose solution with COD value of 38.4 mg L^−1^, an obvious oxidation current step is observed (curve b). If we gradually increase the COD value to 57.6 mg L^−1^ (curve c), 76.8 mg L^−1^ (curve d), 96 mg L^−1^ (curve e), 134.4 mg L^−1^ (curve f), 192 mg L^−1^ (curve g), 288 mg L^−1^ (curve h), 384 mg L^−1^ (curve i), 576 mg L^−1^ (curve j) and 768 mg L^−1^ (curve k), it is found that the oxidation response current increases linearly, suggesting a good linear response.

**Fig. 4:**
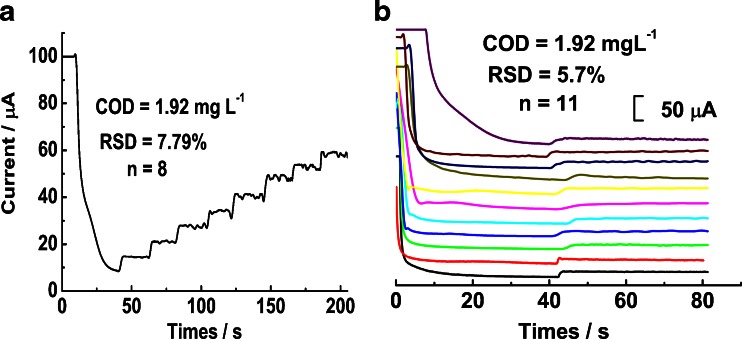
A: The amperometric response of 1.92 mg L^−1^ glucose with successive detections. B: The amperometric response of 1.92 mg L^−1^ glucose to eleven multiple micro-nano Cu-Cos. Other conditions are as in Fig. 2

Further studies by drawing a standard curve demonstrate the oxidation current signal (i, A) is proportional to the concentration of COD (C, mg L^−1^ of O_2_) for glucose solution over the range from 1.92 to 768 mg L^−1^, as shown in Fig. 4B. The linear regression equation is i = 0.888 COD and the correlation coefficient is 0.999. Otherwise, the limit of detection (LOD) was evaluated to be 0.609 mg L^−1^ according to IUPAC regulations (S/N = 3).

### Reproducibility, repeatability and influence of chloride ion

The reproducibility of one micro-nano Cu-Co/gold electrode for successive detections was evaluated as Fig. 4A shown by adding glucose solutions of 1.92 mg L^−1^ glucose 8 times in succession, and a relative standard deviation (RSD) of 7.79 % was obtained, suggesting very good reproducibility. Then, the reproducibility between multiple micro-nano Cu-Co/gold electrodes was tested also using the response signal by glucose solutions of 1.92 mg L^−1^ COD. The value of RSD is just 5.7 % for 11 μ-nano Cu-Co films as Fig. 4B shows, indicative of excellent fabrication reproducibility and detection precision.

The influence of chloride ion on the amperometric detection of COD was also studied. In the presence of 0.02 M chloride ion, the response current signal of glucose solution (COD = 1.92 mg L^−1^) kept unchanged, revealing high tolerance level to chloride ion.

## Analytical application

**Fig. 5:**
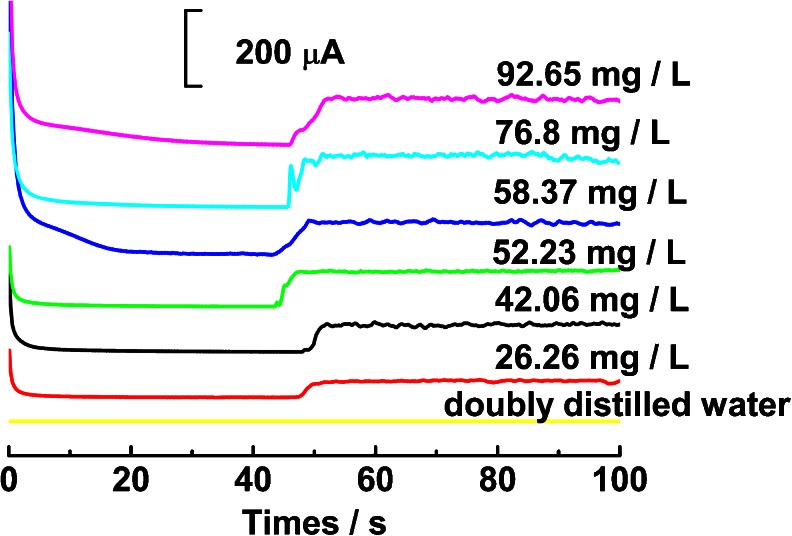
Amperometric response of lake water samples on micro-nano Cu-Co/gold electrode that was prepared at −200 μA for 100 s

**Fig. 6:**
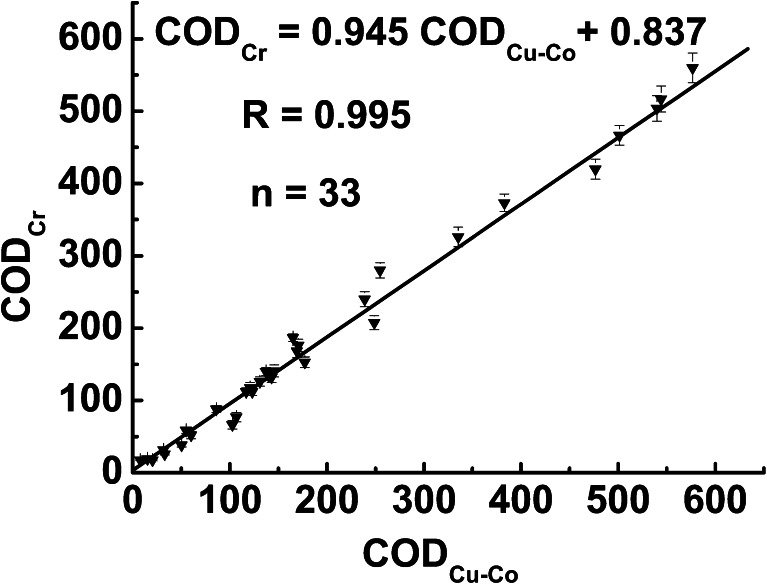
Relationship between COD values of surface water obtained by micro-nano Cu-Co electrochemical sensor and the conventional dichromate method

In order to testify the performance of this sensor in the analysis of real water samples, it was used to detect COD values of surface water samples that were collected from different lakes in Wuhan. Fig. 5 demonstrates the amperometric response of six water samples on the micro-nano Cu-Co/gold surface. The COD concentration of water samples was detected using dichromate method, and the values are 26.26, 42.06, 52.23, 58.37, 76.8, and 92.65 mg L^−1^. It is found that the current response of water sample increases linearly with the COD value, suggesting promising application. Subsequently, the micro-nano Cu-Co/gold was then used in a large number of water samples. For testing the accuracy of micro-nano Cu-Co, the COD value of water samples were also analyzed by the conventional dichromate method. Each sample solution underwent three-parallel detections. Fig. 6 shows the relationship between the results obtained by this new method and by the conventional method for 33 surface water samples. It is found that the COD value detected by the dichromate titration method (indicated as COD_Cr_) has good correlation with that by micro-nano Cu-Co sensor (denoted as COD_Cu-Co_). The linear regression equation is COD_Cr_ = 0.945 COD_Cu-Co_ + 0.837, and the correlation coefficient is 0.995, which indicated that the two methods have good accordance. Therefore, this new electrochemical method using micro-nano Cu-Co is feasible for the detection of COD.

## Conclusion

We report an alloy composite of micro-nano Cu-Co which was facilely fabricated by one-step electrodeposition on gold electrode and then used as sensing materials for the electrochemical detection of COD employing glucose as standard substance. Due to the synergistic effects to oxygen-oxygen band breaking between copper and cobalt, together with high surface roughness and numerous micro and nano particles of the alloy composite, the fabricated electrode displays larger oxidative current response compared to gold electrode modified with copper or cobalt only. This sensing material not only shows the improved sensitivity and long-term stability, but also offers the advantages of simple fabrication process and efficient application in determination of COD in real water samples, which are essential for the COD detection in environmental monitoring. In a word, the present work demonstrates a promising application of intermetallic micro-nano material composite as highly active sensing materials in high-performance electrochemical sensors. Besides, this study also provides a general protocol to fabricate various sensors with intermetallic materials as sensing film for the sensitive detection of glucose and COD and provides a strategy for exploring the properties of intermetallic materials based on the property and interaction of monometallic component.

## Electronic supplementary material

Below is the link to the electronic supplementary material.

ESM 1 (PDF 823 kb)
