# Supplementary material for: Electrochemical tuning of the activity and structure of a copper-cobalt micro-nano film on a gold electrode, and its application to the determination of glucose and of Chemical Oxygen Demand
Source: Mikrochim Acta. 2014 Sep 17;182(3):515–22. doi: 10.1007/s00604-014-1353-z (PMC4298665; doi:10.1007/s00604-014-1353-z)
Supplement: Supplementary file 1 — (PDF 823 kb) [file 604_2014_1353_MOESM1_ESM.pdf]

Electronic Supplementary Material

Electrochemical tuning of the activity and structure of a copper-cobalt micro-nano film on a gold electrode, and its application to the determination of glucose and of Chemical Oxygen Demand

Jinqi Wang[a], Na Yao[a], Mei Li[a], Jia Hu[a], Jianwei Chen[a], Qiaoling Hao[a], Kangbing Wu[a,b], Yikai Zhou[a]*

[a] *MOE Key Lab of Environment and Health, School of Public Health, Tongji Medical College, Huazhong University of Science and Technology, Wuhan 430030, China*

[b] *School of Chemistry and Chemical Engineering, Huazhong University of Science and Technology, Wuhan 430074, China*

*Corresponding author: Phone: 86-27-83692730, Fax: 86-27-83632333, Email: zhouyikaitjmu@163.com

Influence of metal ion concentration on the activity of micro-nano Cu-Co

It has been known that copper is an excellent electrode material for carbohydrates analysis even including COD analysis [1,2]. In this work, copper particles were coated on the gold surface using 0.1 M, pH 4.0 acetate buffer under reduction current of −200 μA and reduction time of 100 s in different-concentrated $CuCl_2$ solution including 20, 24, 26, 28, 29, 30, 31, 32, 34, 36, and 40 mM. Subsequently, the activity of obtained coatings was tested using the oxidation signal of glucose by linear sweep voltammetry (LSV) in 0.1 M NaOH solution. As displayed in Fig. S1, the response current of 19.2 mg L^{-1} glucose significantly increased with Cu^{2+} concentration from 20 to 30 mM and then decreased if further improving Cu^{2+} concentration to 40 mM. Thus, 30 mM Cu^{2+} was employed to prepare sensing film.

In this study, Co^{2+} was added to prepare Cu-Co alloy electrode, remaining the concentration of metal ions as 30 mM. During the co-deposition of Cu^{2+} and Co^{2+}, the

molar ratio of them changed while the total metal ion concentration stays the same. The molar ratio of $CuCl_2$ and $Co(NO_3)_2$ on the catalytic activity of intermetallic composite was studied based on the oxidation signal of glucose in 0.1 M NaOH solution (Fig. S2). Fig. S2A shows the LSV curves on different Cu-Co films for glucose with COD of 19.2 mg L^{-1}, and Fig. S2B demonstrates the influence of different molar ratio of Cu^{2+}, Co^{2+} while the total metal concentration was conserved as 30 Mm. In the absence of Cu^{2+}, the response current for glucose solution was very low (curve a). While gradually improving the Cu^{2+} concentration to 20 mM, the response current increased slowly. If further improving Cu^{2+} concentration to 27 mM, the response current increased linearly. As the concentration of Cu^{2+} beyond 27 mM, the response signal decreased swiftly. This is because that the synergistic effects to oxygen-oxygen band breaking between the copper and cobalt become weaker when the concentration of Cu^{2+} is too high. Clearly, the electrocatalytic activity of Cu-Co film can be tuned by the different concentration ratio of Cu^{2+} and Co^{2+}. Thus, for obtaining high electrochemical activity, Cu-Co sensing film prepared under 27 mM Cu^{2+} and 3 mM Co^{2+} was employed.

Influence of deposition medium and deposition time

Various micro-nano Cu-Co coatings were prepared using 27 mM $CuCl_2$ and 3 mM $Co(NO_3)_2$ in different solution including 0.01 M HCl (pH 2); 0.1 M HAc (pH 2.87); 0.1 M acetate buffer solution with pH of 3.6, 4, 4.6, 5 and 5.6; and 0.1 M $NaNO_3$ (pH 7). After that, the electrochemical activity of resulting micro-nano Cu-Co was analyzed by LSV based on the oxidation peak current of 19.2 mg L^{-1} glucose with reduction current of −200 μA, and reduction time of 100 s. As shown in Fig. S3, the response current of glucose on the surface of micro-nano Cu-Co in 0.1 M NaOH solution greatly increases with pH value from 2 to 4. However, the oxidation current of glucose starts to decrease when the pH value is > 4. The pH value of solution not only affects the mass transport of Cu^{2+} and Co^{2+}, but also influences their electron transfer rate on gold surface. Herein, to achieve high response activity, micro-nano Cu-Co sensing film was prepared in 0.1 M, pH 4.0 acetate buffer to obtain high electrochemical activity.

The influence of deposition time on the electrochemical activity of micro-nano Cu-Co was also studied using 27 mM $CuCl_2$ and 3 mM $Co(NO_3)_2$ in 0.1 M, pH 4.0

acetate buffer with reduction current of −200 μA. The LSV curves of glucose on different micro-nano Cu-Co/gold electrodes are given in Fig. S4A, and the oxidation current as a function of deposition time is displayed in Fig. S4B. When the deposition time extends from 80 to 100 s, the activity of prepared micro-nano Cu-Co toward the electrochemical oxidation of 19.2 mg L^{-1} glucose in 0.1 M NaOH solution remarkably increases, accompanied by notable oxidation current enhancement. However, the oxidation current of glucose on the surface of micro-nano Cu-Co gradually decreases with further extending the deposition time from 100 to 120 s. This is because that the particle size of deposits obviously increases when the deposition time exceeds 100 s. Consequently, the electrochemical activity of micro-nano Cu-Co to the oxidation of glucose also decreases. Thus, 100-s oxidation deposition was used to prepare micro-nano Cu-Co sensing film for getting the best sensitivity.

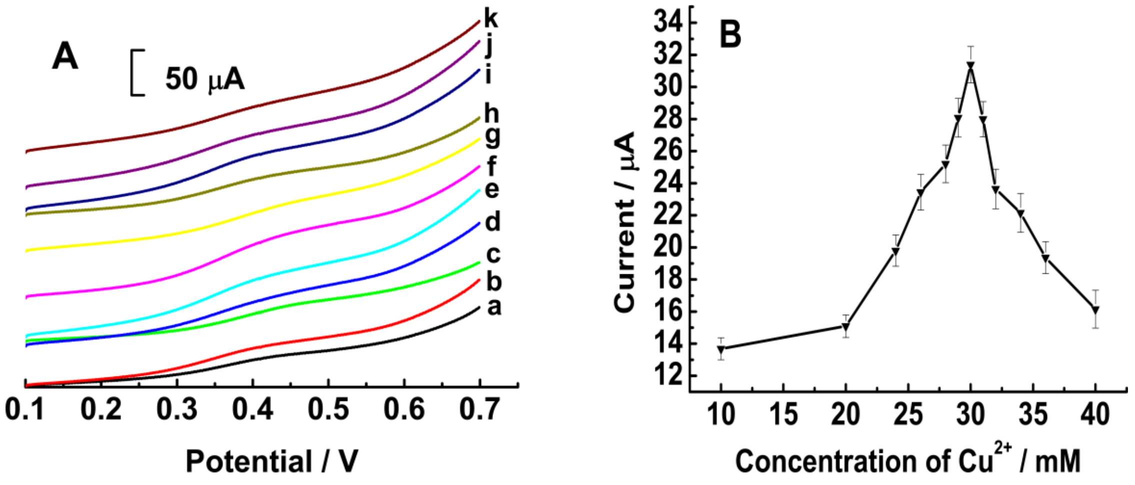

Fig. S1 A: The LSV curves of 19.2 mg L^{-1} glucose on different copper films that deposited at different concentrations of Cu^{2+}, including 20 mM (a), 24 Mm (b), 26 Mm (c), 28 mM (d), 29 Mm (e), 30 mM (f), 31 mM (g), 32 Mm (h), 34 mM (i), 36 mM (j) , and 40 Mm (k). B: Variation of the oxidation peak current of glucose as the different concentration of Cu^{2+}. The error bars represent the standard deviation of repetitive measurements (n = 3).

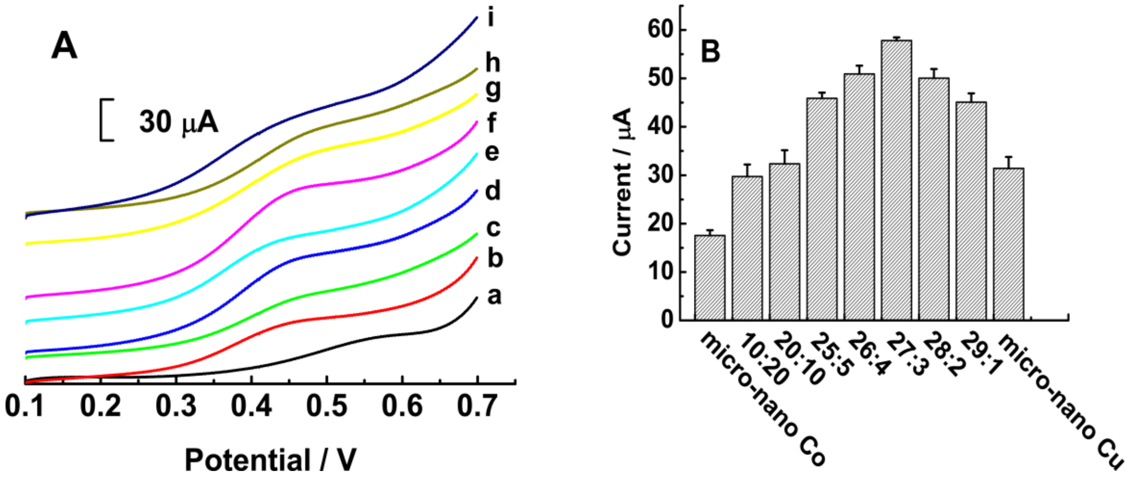

Fig. S2 A: The LSV curves of 19.2 mg L^{-1} glucose on different films that deposited at different proportion concentration of Cu^{2+}, Co^{2+} including 30 Co (a), 10/20 (C_{Cu}/C_{Co}) (b), 20/10 (c), 25/5 (d), 26/4 (e), 27/3 (f), 28/2 (g), 29/1 (h), and 30 Cu (i). B: Variation of the oxidation peak current of glucose as the different concentration of Cu^{2+}, Co^{2+}. Other conditions are as in Fig. S1.

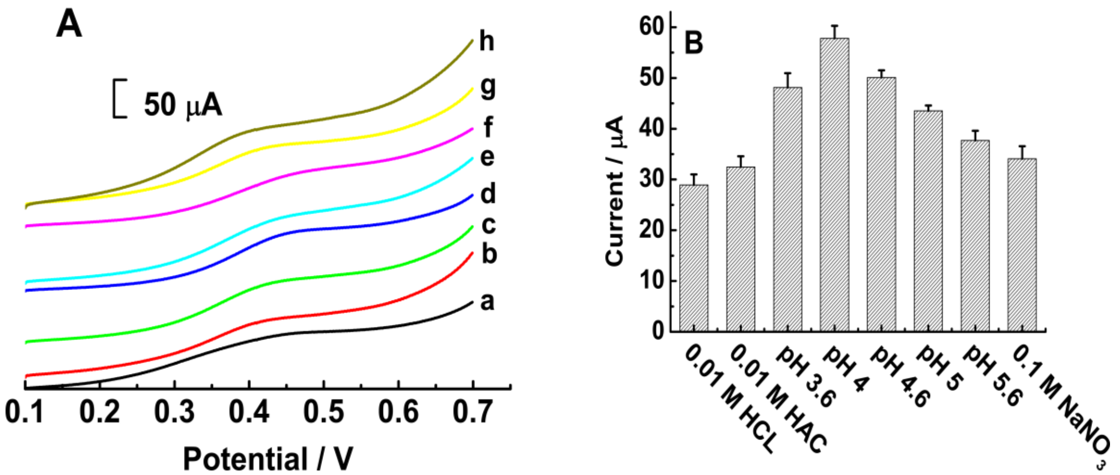

Fig. S3 A: The LSV curves of glucose on micro-nano Cu-Cos that prepared at pH 2.0 (a), pH 3.0 (b), pH 3.6 (c), pH 4.0 (d), pH 4.6 (e), pH 5.0 (f), pH 5.6 (g), and pH 7.0 (h) solutions. B: Variation of the oxidation peak current of 19.2 mg L^{-1} glucose at different pH-valued solution. Other conditions are as in Fig. S1.

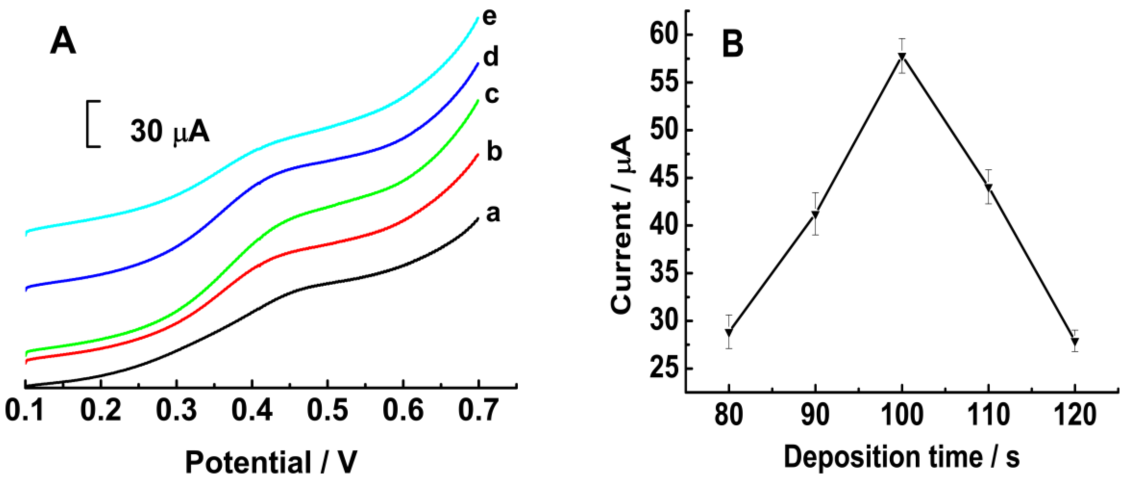

Fig. S4 A: The LSV curves of 19.2 mg L^{-1} glucose on micro-nano Cu-Cos that deposited at 200 μA for 80 s (a), 90 s (b), 100 s (c), 110 s (d), and 120 s (e). B: Variation of the oxidation peak current of glucose as the reduction time (B). Other conditions are as in Fig. S1.

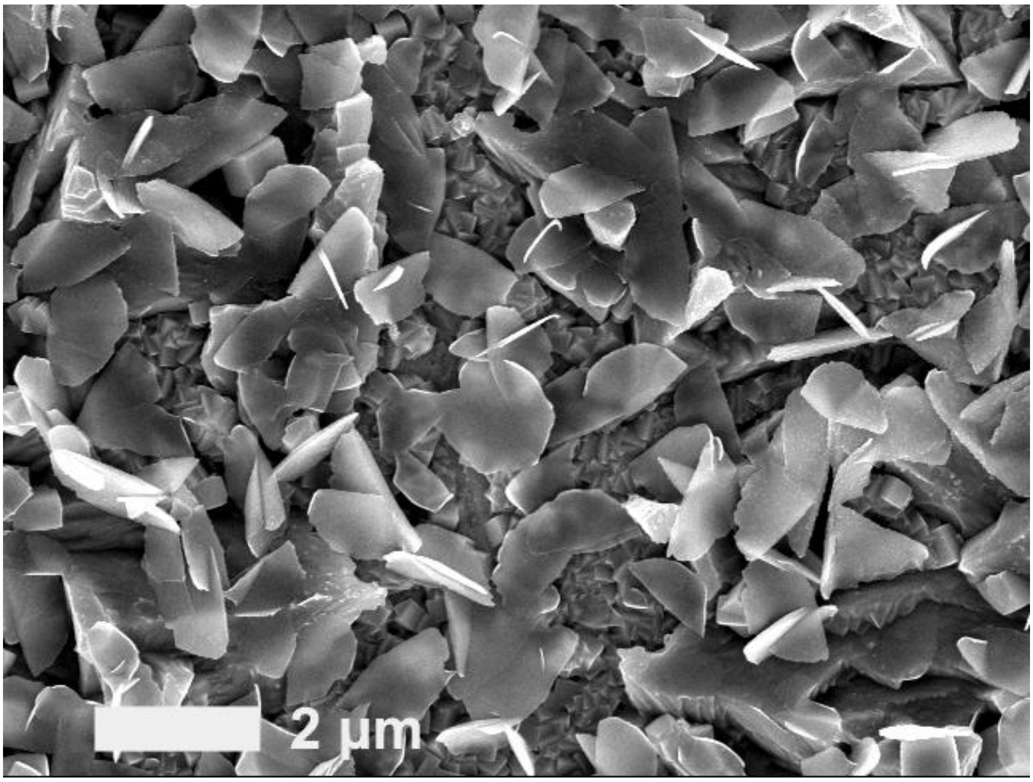

Fig. S5 SEM images of micro-nano Cu deposited on gold surface at current of − 200 μA.

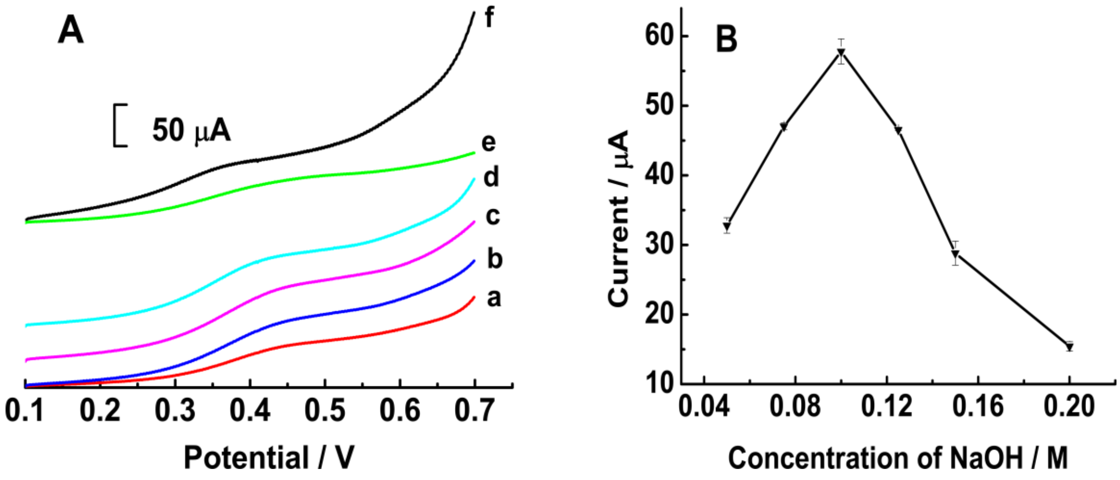

Fig. S6 A: The LSV curves of 19.2 mg L^{-1} glucose on micro-nano Cu-Cos in different concentration of NaOH solution including 0.05 M (a), 0.075 M (b), 0.1 M (c), 0.125 M (d), 0.15 M (e) and 0.2 M (f). B: Variation of the oxidation peak current of glucose as the different concentration of electrolyte. Other conditions are as in Fig. S1.

References

1. Wang F, Liao Y, Ren Y, Chen Z (2010) A novel method for D-arabinitol determination based on a nano-structured sensing film by one-step electrodeposition. Microchimica Acta 170 (1-2):9-15

2. Shi H, Zhang Z, Wang Y, Zhu Q, Song W (2011) Bimetallic nano-structured glucose sensing electrode composed of copper atoms deposited on gold nanoparticles. Microchimica Acta 173 (1-2):85-94
